# The Role of Nutritional Support in Cured/Chronic Patients

**DOI:** 10.3390/nu12103167

**Published:** 2020-10-16

**Authors:** Giovanni Rosti, Fabrizio Romano, Simona Secondino, Riccardo Caccialanza, Federica Lobascio, Ornella Carminati, Paolo Pedrazzoli, Paolo Tralongo

**Affiliations:** 1 Medical Oncology, Fondazione IRCCS Policlinico San Matteo, 27100 Pavia, Italy; s.secondino@smatteo.pv.it (S.S.); p.pedrazzoli@smatteo.pv.it (P.P.); 2 Medical Oncology Department, Ospedale Umberto 1-RAO-Siracusa, 96100 Syracuse, Italy; romano@raosr.it (F.R.); tralongo@raosr.it (P.T.); 3 Clinical Nutrition and Dietetics Unit, Fondazione IRCCS, Policlinico San Matteo, 27100 Pavia, Italy; r.caccialanza@smatteo.pv.it (R.C.); f.lobascio@smatteo.pv.it (F.L.); 4 Medical Oncology, Department of Oncology and Hematology, AUSL Romagna, 48100 Ravenna, Italy; ornella.carminati.mail@gmail.com; 5 Department of Internal Medicine and Medical Therapy, University of Pavia, 27100 Pavia, Italy

**Keywords:** cancer survivors, diet, nutritional care

## Abstract

Improvements in Clinical Oncology, due to earlier diagnoses and more efficient therapeutic strategies, have led to increased numbers of long-term survivors, albeit many with chronic diseases. Dealing with the complex care needs of these survivors is now an important part of Medical Oncology. Suitable diet and physical activity regimes will be important in maintaining their health. This paper will review what we know and what we can do in the near future for these patients.

## 1. Cured and Long-Term Cancer Patients

Due to improved screening programs and therapeutic advances, long-term survival is increasingly common in patients diagnosed with cancer [1,2]. The number of patients with a history of cancer in Italy rose from about 2 million in 2006 to over 3 million in 2016 [3], and by the end of 2020 we expect to have about 4.5 million cancer patients. The Italian data are in line with the rest of the world: the number of cancer survivors in the US, for example, has increased from 3 million in 1970 to about 15 million 2012, representing 5% of the total population. [2].

The risk of death from a specific cancer is highest in the initial years after diagnosis and tends to decrease progressively thereafter. In some cases, it becomes negligible and such patients reach a life expectancy that corresponds to that of the general population of the same age and gender [4,5]. For example, epidemiological data indicate that excess mortality falls to zero after 8 years in colorectal [6,7] and invasive cervical cancer [8]. Five-year survival is now over 95% for thyroid and testicular cancers among adult Italian patients. For patients with these types of cancers, 10-year survival reached approximately 90% [9] during the period 2000–2004, suggesting a very good prognosis and a similar long-term life expectancy to that of the sex- and age-matched general population.

Survival is also expected to increase for other types of cancer in the near future through the use of personalized treatments based on a better understanding of biology and the potential responses to therapy.

The use of the term “*cured*” for some cancer patients has been proposed in view of increasing survival rates in some cancers [10]; as reported in the Siracusa Charter “the word *cured* refers to complete clinical remission of a cancer, regardless of the presence or absence of delayed treatment sequelae”. In order to correctly apply the word “cured,” it is necessary that the time from diagnosis of cancer must be such that the patient’s risk of death does not exceed that of a sex- and age-matched general population. At present the word “*cured*” cannot be used for all types of cancer, because cancer is a highly heterogeneous group of diseases with varying biological characteristics, clinical expressions, natural history, response to treatment and outcomes.

## 2. General Dietary Information in This Setting

**Figure 1:**
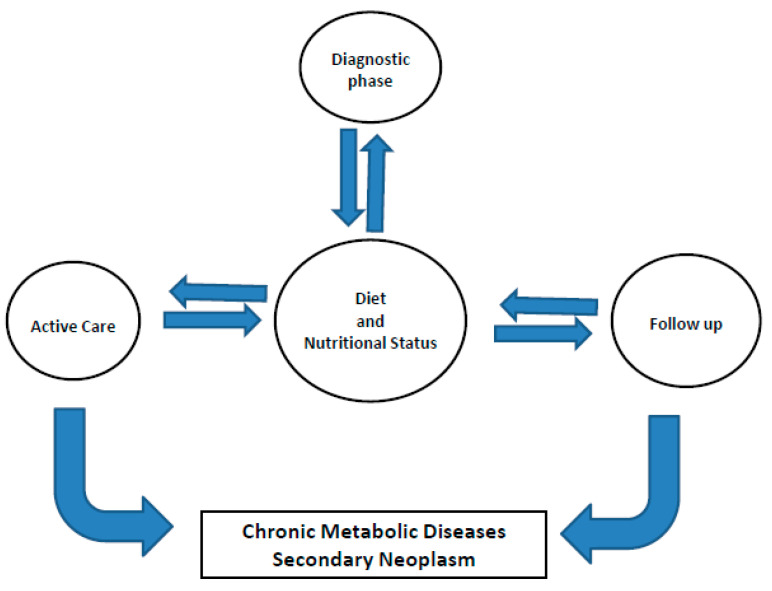
Shows the relationship amid diet/nutritional status and phases of the “cancer journey”.

Diet and nutritional status are crucial to diagnosis, the choice of an appropriate therapeutic approach and, more than ever before, to the follow-up of active care (Figure 1). During the last decade, much of the research in the field of diet and cancer was aimed at identifying some specific components of the diet such as fibers, individual nutrients or phytochemicals as possible causes of the increase or decrease in cancer incidence: this can be considered as reductionist research strategy.

Individual nutrients or phytochemicals introduced into the diet have only a modest effect on cancer incidence. An integrated model of diet and exercise is required to reduce the DNA mutations that accrue during carcinogenesis. Homeostasis is maintained through complex interactions between neurological, endocrine, immunological and behavioral processes. Diet and nutrition are environmental variables and a varied diet helps maintain homeostasis. Modern techniques in agriculture, food preservation and storage, together with efficient, low-cost transport, have enabled a varied and healthy food supply in many nations. Despite this, disparities and economic barriers persist within countries, and are accompanied by the failure of many governments to formulate and implement strategies aimed at promoting healthy lifestyles. Currently, a large segment of the global population consumes sophisticated, industrially produced foods that are rich in refined carbohydrates and fats but relatively poor in nutrients from fruits, vegetables, legumes and whole grains. This, combined with today’s sedentary lifestyle, contributes to epidemic obesity. This is likely to compromise homeostasis, leading to the development of chronic diseases, cardiac problems and cancer [11,12].

It is therefore reasonable to believe that the incidence of these comorbidities in cured/chronic patients can be reduced by lifestyle changes; in fact, it has been shown that those who have had an oncological diagnosis are much more predisposed and motivated to change their habits [13,14].

Cancer survivors have both an increased risk of secondary neoplasms and a higher incidence of comorbidity than the general population [15]. An increased incidence of cardiovascular disease, diabetes and osteoporosis is documented in long-term patients, influenced by genetic and environmental factors which contribute significantly to increased morbidity and oncological recurrence. The elderly population benefits most from an improved diet and physical activity, demonstrating reduced fragility and improved vitality. Elderly cancer survivors have improved their diet and their physique. The available evidence supports initiatives aimed at improving diet, physical activity and therefore quality of life in long-term patients [16]. Data processing is likely to assist research into how diet can influence the health status of these patients. Reviewing the literature can help define appropriate clinical practice and social policies [17].

## 3. Hot Topics: Fibers, Protein and Carbohydrates

Proteins, carbohydrates and fats provide energy value to the diet, each of these components is available in a wide variety of foods. As already mentioned, long-term patients are at high risk of developing chronic illnesses like cardiovascular diseases. These individuals, and particularly those who are overweight, should be prescribed an adequate, but not excessive, intake of fat, protein and carbohydrates [18]. The American Heart Association (AHA), recommends a dietary composition for the adult population as follows: fat: from 20% to 35% energy (AHA: 25–35%), carbohydrates: from 45% to 65% energy (AHA: 50–60%); and protein: from 10% to 35% energy (at least 0.8 g/kg). Several studies have examined the relationship between fat intake and survival after diagnosis of breast cancer, but the results of some of these studies, aimed at assessing dietary composition and survival, appear controversial.

Two large randomized controlled trials have assessed the relationship between low-fat diet and outcome in patients with early-stage breast carcinoma. The WINS study tested a low-fat diet (less than 15% energy) in 2437 postmenopausal women with early-stage breast cancer, showing a limited effect on recurrence-free survival. On average, women in the intervention arm reduced fat intake to 20% energy in the first year and the intervention resulted in a 24% reduction in new breast cancer events, mainly in women with estrogen-negative tumors. Note, as previously described, women assigned to the low-fat diet study arm lost an average of 2.7 kg during the study, thus confusing whether the reduction in breast cancer events was due to restriction of fat in the diet or reduction in body weight [19]. The WHEL study tested the effect of a diet low in fat (targeting 20% energy intake) and very rich in vegetables, fruit and fiber, on cancer outcomes in 3088 pre and postmenopausal women considered to be cured of breast cancer, and who were followed for an average of 7.3 years. After 4 years, women in the intervention group reported a reduction in fat intake (from 31.3% at enrolment to 26.9% of energy intake), but survival did not differ between the two study arms. In particular, women in the WHEL study intervention group showed no weight loss, in contrast to the low-fat diet intervention group in WINS [20].

Low-energy foods can induce satiety, and thus promote the achievement or maintenance of an adequate body weight [21]; whole fruit adds more fiber and fewer calories to the diet than fruit juices. As noted above, the results of more recent studies suggest that a diet rich in fruits and vegetables is associated with increased overall survival after cancer diagnosis and treatment. Typically, this dietary model contains fish and poultry instead of red or processed meat, low-fat dairy products instead of full-fat, whole grains instead of refined cereal products, nuts and olive oil instead of other sources of fat [22]. A study in patients cured of colon cancer showed that a Western diet, characterized by a high intake of meat and added sugars, was negatively associated not only with cancer related survival, but also with reduced overall survival [23]. In observational studies that examined the relationship between intake of fruit and vegetables and the risk of recurrence of breast cancer, mixed results were reported [24]. The WHEL study tested the effect of a very high dietary intake (7.3 portions/day) of vegetables, fruits and fiber, on the risk of recurrence and overall survival in early-stage breast cancer survivors. After 6 years, it had increased to an average of 9.2 portions per day compared to the control group’s average of 6.2 portions per day; recurrence-free survival did not differ between the two study arms. However, basal serum estrogen levels were independently associated with poor prognosis and the protective effect of the diet was observed in the subgroup of women who reported no hot flushes at enrolment or higher estrogen levels [20]. These results suggest that a diet rich in vegetables, fruits and fiber affects the hormonal state and therefore the prognosis. In addition, longitudinal carotenoid exposure was associated with breast cancer-free survival, regardless of study group assignment. The quality of the diet prior to oncological diagnosis may be more important than short-term post-diagnosis dietary changes. Some studies have assessed the association between diet and ovarian cancer survival. A higher pre-diagnosis intake of vegetables, especially yellow and cruciferous vegetables, was associated with a longer survival in these studies [25].

A single observational study on diet and progression after diagnosis of prostate cancer, showed that those who had consumed more tomato sauce had a longer survival [26]. Currently, public health recommendations indicate 2 or 3 cups of vegetables and 1.5 or 2 cups of fruit per day for adults. Colored choices such as dark green and orange vegetables are good sources of nutrients, and are potentially healthy. Fresh, frozen, canned, raw, cooked or dried fruits and vegetables all contribute nutrients and other biologically active components to the diet. Cooking vegetables and fruit by methods such as microwave or steam cooking, are preferable to boiling in large amounts of water, by preserving the bioavailability of water-soluble nutrients and improving the absorption of others. For example, carotenoids are better absorbed from cooked vegetables than from raw vegetables. There is no evidence that organically grown fruits and vegetables have a higher content of potential anti-tumor components.

## 4. Dietary Supplements: Vitamins, Minerals and other Nutrients

Food supplements include vitamins, minerals, herbs/botanicals, and amino acids. Around 52% of U.S. adults report using dietary supplements, increasing to between 64% and 81% among long-term patients. A systematic review indicates that 14% to 32% of long-term survivors take supplements after diagnosis, and this is most prevalent in breast cancer patients lowest in those with prostate cancer [27]. Often the choice to use a dietary supplement is dictated by the idea that it can help reduce the risk of recurrence and thus improve survival.

Observational studies suggest it is unlikely that dietary supplements can improve prognosis or overall survival after cancer diagnosis and can actually increase mortality. A 2006 metanalysis shows no association between antioxidants or vitamin A supplementation and mortality from all causes in cancer patients, although the authors noted that this result was based on a limited number of tests, particularly high-quality tests [28]. The use of multivitamins or vitamins E or C was not associated with cancer death protection in a cohort of 77,719 Washington state residents followed for a period of 10 years [29]. In two large observational studies, the use of a full range of dietary supplements or multivitamins in particular, was not associated with a reduction in recurrent breast cancer, specific breast cancer mortality or general mortality among women diagnosed with early breast cancer [30,31].

It may be better to encourage breast cancer survivors to derive appropriate nutrients from a balanced diet and to use supplements only when this fails to correct a deficiency. Emerging evidence suggests that increased nutrient intake, especially from sources other than food, can be harmful rather than helpful. This requires tactful conversations between doctor and patient in order to place the prescription of a supplement second to an actual need.

## 5. Diet and Nutritional Therapy

Obesity, metabolic syndrome, declining fitness and their sequelae are common in long-term patients. When we consider the additional risks related to the long-term toxicity of treatments, access to safe and effective interventions is essential to promote health and quality of life. To date, many care and rehabilitation programs have been organized according to standardized models and few attempts have been made to integrate targeted, individualized measures into the care program [32].

## 6. Maintaining Body Weight

Cancer and cancer treatments may lead to modification in terms of body weight, which may be long-lasting after cancer therapy has been completed (either by surgery, pharmacologic treatment, radiation or combinations of these) [33]. When cancer treatment is over, weight gain or loss should be managed with a combination of dietary, physical activity, and behavioral strategies.

### 6.1. Weight Loss

For patients who become underweight, the overall goals for nutritional care are to prevent or reverse nutrient deficiencies to preserve lean body mass, to minimize nutrition-related side effects (reduced appetite, changes in smell and taste, nausea and/or vomiting) so maximizing quality of life. [34] and, in addition, to increase energy intake to exceed energy expended. During cancer treatment especially, those patients of normal or lower body mass index and those with head and neck, lung, and some gastro-intestinal tumors are most at risk of weight loss stemming from undernutrition and poorer quality of life [35].

For those at risk of unintentional weight loss, interventions should focus on increasing food intake to achieve a positive energy balance and therefore increase weight. For added calories, the consumption of more nutrient and energy dense foods (yogurt, dried fruit, cheese, legumes and eggs) is recommended [36]. Moreover, smaller, more frequent feedings can prove helpful in obtaining adequate energy. In cases of long-lasting mouth sores, which may impede the easy consumption of foods that are abrasives, soft bland foods are better tolerated. To date, weight loss in survivors without active cancer has not been reported as a negative or positive predictive factor itself regarding survival, although there is a paucity of data in the literature.

### 6.2. Weight Gain

At the other end of the spectrum weight gain can undermine the health and survival of cancer survivors. Pathologic overweight gain and obesity, are well known and established risk factors for some cancers like endometrium, breast, and others, so at the time of diagnosis several patients will fall into these two categories especially in western regions of the world [37]. There are some data on the negative association of obesity at diagnosis with cancer survival for the majority of cancers, including hematologic neoplastic diseases [38].

A substantial proportion of survivors (up to 70%) have a BMI > 25 (data are mainly for breast and prostate cancer) [39]; it should be noted that some treatments lead to increased body weight (i.e., adjuvant therapies for breast cancer or hormonal deprivation in prostate cancer). A relationship between diet and cancer mortality, independent of weight gain, has been reported. In particular, in a meta-analysis involving more than 200,000 cancer survivors from 117 studies, higher intakes of vegetables and fish were inversely associated with overall mortality while higher alcohol consumption was positively associated with overall mortality [40]. However, limiting analysis to weight gain, an increase in BMI post-diagnosis of at least 0.5 units significantly increases the risk of cancer recurrence and mortality from all causes.

Some other studies on modern short adjuvant programs in operable breast cancer do not support a relationship between weight change and survival [41]. In contrast, in a recent paper, Troeschel et al. [42] reported that there was a significant positive association between post-diagnostic obesity and prostate cancer mortality among men with low-risk tumors (defined as T1/T2 or Gleason 7 tumors). This highlights the importance of not only obesity avoidance, but also of weight maintenance, in this wide population of potentially curable disease.

So, weight management is a high priority for cancer survivors. While modest rates of weight loss can be achieved by portion control and by substituting low-energy foods (i.e., whole grain and water-rich vegetables or fruits), for foods higher in calories, more structured intensive programs including exercise may yield better results: exercise has been shown to be a strong predictor or weight loss in cancer survivors, so multifaceted programs need to be implemented.

## 7. Constipation

Constipation is a common problem for many cancer survivors; the causes are various, from opioid consumption (some survivors require analgesic therapy even for long time after cure), previous therapy with vinca alkaloids, decrease intake of fibers or a combination of such factors [43].

Prevention is the best way to approach the problem, and survivors are encouraged to have a diet high in fibers and liquids. Stool softeners and laxatives are also indicated in some circumstances such as senna of psyllium methylcellulose. In unsuccessful cases, polyethylene glycol or lactulose may be necessary. Diet is important and diet counselling is advised to improve the program to prevent constipation, especially by improving fiber intake [44].

## 8. Diet and Fatigue

Persistent fatigue is one of the most prevalent and burdensome late-term effects of cancer treatment, particularly in the elderly [45]. It can be described as a subjective sense of physical, emotional and/or cognitive tiredness or exhaustion that is not proportional to recent activity and interferes with normal functioning. Its etiology is currently unknown; however, research suggests that peripheral pro-inflammatory markers including cytokines and C-reactive protein are consistently elevated in fatigued cancer survivors [46]. Fatigue can last for years after the end of therapy [47].

In a small randomized French study [48], a diet rich in fruit, vegetables, whole grains, and omega-3 fatty acid-rich foods, (named the fatigue reduction diet—FRD), improved fatigue and sleep compared to an attention control named the general health curriculum. In another report on breast cancer survivors, fatigue was positively associated with % of kcal/day fat intake (r = 0.31, *p* < 0.05) and inversely related to fiber g/day (r = 0.38, *p* < 0.05) and carbohydrate g/day intake (r = 0.31, *p* < 0.05). Mean fatigue was greater for participants eating <25 g/day of fiber compared with >25 g/day of fiber (15.7 ± 10.8 versus 6.4 ± 3.7, *p* < 0.005) [49]. A large systematic review evaluated the role of nutrition and exercise in prostate cancer survivors, but failed to show any impact of nutrition therapy. Large randomized clinical trials are urgently needed to resolve this question [50].

## 9. Diet and Nausea

**Figure 2:**
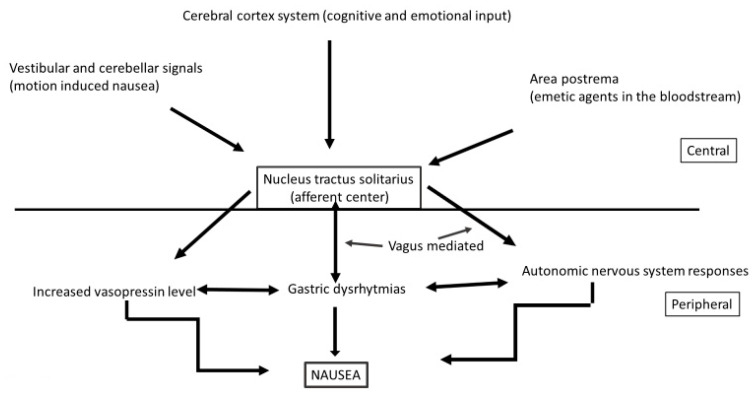
Pathophysiology of nausea.

Nausea and vomiting are among the worst side effects of chemo and radiotherapy, even though modern prophylactic pharmacologic interventions have reduced vomiting to below 10% in patients receiving highly or moderately emetogenic treatments (compared to nearly 100% three decades ago). Nausea, however, still remains a problem [51]. The loops involved in vomiting are well understood One involves serotonin where stimuli travel through the vagus nerve from the gut to the so-called chemoreceptor trigger zone in the *area postrema* and activate the “vomiting centre’’ [51]; this mechanism is involved in early vomiting and nausea. The other loop involves NK-1 receptors which are located mainly in the central nervous system and are responsible mainly for late nausea. Probably some mechanisms or pathways or receptors are not fully known or understood, which would explain why nausea might still be a problem. Possible causes of nausea are illustrated in Figure 2.

In cancer survivors, nausea is not a common event and usually does not differ from nausea in individuals without a history of cancer; nevertheless, in cases of persistent nausea after active treatment is over, metoclopramide or similar agents may be used, but limiting the dose to no more than 30 mg per day due to the possible extrapyramidal side effects and for no more than thirty days [52]. There are no indications for other drugs like NK-1 receptor antagonists (aprepitant) of serotonin receptor antagonists (setrons). A more difficult situation arises when patients are undergoing adjuvant treatment as is the case with imatinib for GIST or chronic myelogenous leukemia, or temozolomide for brain tumors. In these situations where the treatment may last for months or years there are no clear indications: metoclopramide may be useful, but with the limitation mentioned above; perhaps non-pharmacologic products may have a role.

One of the most studied herbs is ginger (Zingiber officinale) which has been used for centuries both as a spice and an herbal medicine to treat a variety of primary gastrointestinal ailments. Even if the exact dosage is not known, usually 1–2 g of ginger daily may lead to a benefit on nausea. The mechanism of action of ginger is increasing gastric tone and motility due to its anticholinergic and anti-serotoninergic actions [53]. Acupuncture and acupressure are two techniques commonly used in traditional Chinese medicine to treat nausea and vomiting; during acupuncture, thin needles are inserted into specific points on the body; acupressure aims to stimulate the same points of the body, but uses pressure instead of needles to do so. Both techniques stimulate nerve fibers, which transmit signals to the brain and spinal cord, and these signals are thought to have the ability to decrease nausea [54]; better integration of these techniques with classic medicine is needed.

## 10. Change in Lifestyle

There is a considerable literature on lifestyles intended to prevent neoplastic disease mainly breast and colorectal cancer [55], but far less data on beneficial lifestyle changes for cancer survivors. Quitting cigarette smoking is the most important change in lifestyle regarding general health, and not only cancer development. Abstention from tobacco use may have an impact on secondary cancer or recurrence, but also on overall mortality due to the aggravation of ageing conditions (e.g., coronary disease or COPD) [41]. It has been shown that depressed smokers are 40% less likely to quit, with a resulting higher risk of overall mortality [41]. The same is true for alcohol consumption: it has been reported that patients cured of their head and neck cancer, who continue drinking alcohol, have a nearly four-fold increase in the development of secondary primary disease. This is also seen in cardiovascular, pulmonary and other alcohol-related diseases [53].

As regards diet, eating healthy foods is highly recommended in survivors. Consumption of red and processed meat and salty foods should be limited. Several published manuscripts have reported a positive correlation between the consumption of sugar and cancer progression [56]. Diets should rely on unrefined plant food such as fruits, vegetables and whole grain, with a limited amount of fat and simple sugars [36]. Cancer survivors have a significant lower mean Heath Eating Index (HEI)-2010 compared to non-cancer individuals [57]. Several interventional studies have tested the feasibility and potential benefits of exercise in cancer survivors; data from 85 randomized trials [39] demonstrated that exercise could safely be performed both during and after treatment. Exercise led to significant improvements in aerobic fitness and strength in both settings and increased flexibility and physical functioning post-treatment. Anxiety, depression, fatigue, body image, and quality of life improved, even if not all studies were consistent with this finding.

Major guidelines, such as those of the American Cancer Society and the American College of Sports Medicine, recommend that cancer survivors should incorporate moderate-intensity physical activity into their daily lives; for example > 150 min/weeks of moderate intensity, aerobic exercise and/or walking is safe for the majority of survivors [58]. The ESMO (European Society for Medical Oncology) recommends minimizing the amount of time engaged in sedentary behaviors, such as sitting for long periods in front of a computer or television set. Instead, they suggest engaging in more active alternatives while at home or at work: for example, instead of taking the lift, use the stairs [59] (www.esmo.org). So, apart from dietary information and counselling, our patients who fought and overcame the hurdle of cancer need to try and to change their lifestyle [41]. We, as Doctors, have to continue to be at their side, as we have been during the journey of therapy: this is a must and this is our job.
